# Effects of pressure on the survival and viability of cancer cells *in vitro*: An analytical study

**DOI:** 10.1371/journal.pone.0311685

**Published:** 2025-02-25

**Authors:** Mohsin Ali Khan, Zaw Ali Khan, Ishrat Husain, Shivbrat Upadhyay, Sarina Zehra, Rumana Ahmad

**Affiliations:** 1 Department of Research & Development, Era University, Lucknow, UP., India; 2 Department of Biochemistry, Era’s Lucknow Medical College & Hospital, Era University, Lucknow, India; 3 Department of Biotechnology, Era’s Lucknow Medical College & Hospital, Era University, Lucknow, India; King Abdulaziz University, SAUDI ARABIA

## Abstract

Intense cancer research is underway to discover possible therapies but no major breakthrough appears to be in sight in terms of its cure when diagnosed late. The cytostatic and growth inhibitory effect of high pressure on cells is well documented. In the present study, two cancer cell lines *viz*. MDA-MB-231 (breast carcinoma) and A549 (lung carcinoma) and one normal cell line (Vero) were subjected to increased pressure of 18 psi in a specially constructed pressure chamber. It was found that a pressure of 18 psi induced a significant change in the growth parameters of both cancer cell lines *versus* normal cells. Exposure to increased pressure greatly increased the proportion of MDA-MB-231 cells in the S phase while concurrently reducing the number of cells in the G0/G1 phase as compared to their untreated counterparts. SEM and AFM analysis revealed presence of characteristic ‘pores’ and ‘pits’ on the cell surface of pressure-treated *versus* untreated cancer cells. TEM analysis also revealed significant intracellular differences between pressure-treated and untreated cancer cells. Hyperbaric nitrogen therapy is proposed as a novel cancer-treatment modality involving administration of N_2_ at the tumor site in murine models of breast and lung cancer. This would eventually pave the way for development of a device effective treatment strategy for human tumors in future.

## Introduction

Pressure defined as force per unit area, is an important thermodynamic variable which ranges from 14.5 psi at the surface to 15954.2 psi in the deepest ocean trench. Since cancer cells are characterized by loss of control on cell cycle, it is hypothesized that increased pressure may slow down the rate of cancer cell division. As per the latest cancer facts and figures for 2022 released by American Cancer Society, there were 20 million cancer cases diagnosed and 9.7 million cancer deaths in the United States [1]. The number of cancer cases is predicted to increase to 35 million by 2050, based solely on the projected population growth [2]. Amongst cancers, lung cancer is the most commonly diagnosed cancer and the leading cause of cancer death worldwide, with almost 2.5 million cases (1 in 8 cancers) and 1.8 million deaths (1 in 5 deaths). Lung cancer is also the most commonly found cancer in males followed by prostate, colorectal and stomach cancers while liver, colorectal and stomach cancers cause the most deaths. In females, breast cancer ranks first for both incidence and mortality, followed by lung, colorectal, and cervical cancers.

Any treatment modality that selectively targets cancer cells with minimal effects on surrounding normal cells can become an ideal treatment for cancer. Many anticancer approaches and agents have been developed, but all current strategies face stiff challenges on account of drug resistance, low therapeutic efficiency, and cancer cell selectivity. As opposed to the conventional cancer treatment methods like chemo-and radiotherapy, that have a number of side effects besides their anticancer action, several non-conventional methods of cancer treatment have been developed that have several advantages over the non-conventional methods and are currently in vogue. The best known amongst them are those based on non-thermal atmospheric-pressure plasmas (NTAPP) [3] like dielectric barrier discharge plasma (DBDP) and nitric oxide-plasma activated water (NO-PAW) [4]. Yet another one is hyperbaric oxygen therapy (HBOT) [5].

A recently developed innovation known as cold atmospheric-pressure plasmas (CAPs), also known as non-thermal atmospheric-pressure plasmas (NTAPP) have found several biomedical applications like wound sterilization and healing [3]. Plasma may be described as partially ionized gas containing a quasi-neutral mixture of charged species and radicals. NTAPP can be generated using three kinds of devices *viz*. plasma needle, plasma jet, and dielectric barrier discharge (DBD) [6]. The gases that have been used for NTAPP include inert gases like nitrogen and helium. A recent innovation has involved the inclusion of oxygen or even air (which essentially contains nitrogen and oxygen) to an inert gas for improving the overall efficiency and potency [7]. In recent times, several studies from different groups have independently reported the potential of NTAPP generated from various devices in selectively targeting cancer cells [7–10] and inducing programmed cell death [11–18] *in vitro*. Still others have reported the differential effect of NTPP on cancer *versus* normal cells [9, 19–21]. A few studies have also reported the efficacy of NTAPP in reducing tumor size of xenograft mouse models *in vivo* [22, 23]. However, the mechanism by which NTAPP induces apoptosis in cancer cells has not been well-defined. ROS and RNS are implicated as the main effectors of apoptosis, though this needs to be investigated in detail [3]. It is obvious from the above account that NTAPP has the potential to develop as an effective anticancer therapy in future.

HBOT, involving administration of pure oxygen at pressure higher than the normal atmospheric pressure, is a relatively new treatment modality in cancer and acts as an adjuvant to chemotherapy and radiotherapy [24, 25], although its effect on malignancy remains uncertain [26]. HBOT has proved to be beneficial in local tumor control in case of cancers of head and neck and cervical cancer but not in other cancers [27]. HBOT may exert its antitumor effect through several mechanisms. Since oxygen dissolution increases at higher pressure, more ROS are generated which might have a deleterious effect on tumor cells due to enhanced DNA damage and apoptosis [28–35]. Additionally, high pressure constricts blood capillaries and arterioles, reducing the volume of blood supply to the tumor microenvironment, thus restricting glucose and micronutrient supply to the rapidly proliferating cells within the tumor microenvironment, causing them to die or stop proliferating. It has been demonstrated that there is low oxygen tension within the tumor microenvironment which facilitates angiogenesis and tumor growth [36, 37]. The net oxygen balance remains positive during HBOT which eliminates hypoxia. Change in extra-vascular pressure is known to cause an alteration in blood flow volume and tissue perfusion. Since oxygen at normal pressure fails to diffuse to the inner core of the tumor, the tumors fail to respond adequately to chemo [38] and radiotherapy due to decreased formation of ROS. However, a few studies have also demonstrated that HBOT therapy may have cancer-promoting effects [26, 39] and that the increase in ROS generation may harm the normal cells of the body, thus causing further complications.

Still more confounding are the studies reporting a positive correlation between decreased pressure and low incidence of cancer. Oxidative DNA damage plays a prominent role in the pathogenesis and exacerbation of many diseases including cancer. The relation between elevation and barometric pressure, and hence oxygen, is roughly linear at habitable altitudes [40–42]. Partial pressure of oxygen is reduced to 88.7% at 1,000 m, 78.5% at 2,000 m, and 69.2% at 3,000 m [43]. The dynamics of relation between atmospheric pressure (and hence, oxygen) is assessed by understanding the association between elevation and carcinogenesis. Numerous reports and observations of lower cancer rates at higher elevations have appeared in the literature in the last four decades [43–49]. Of particular relevance are the studies of Weinberg, Brown and Hoel (1987) and Van Pelt (2003) which have suggested intracellular formation of ROS as a possible explanation. Importantly, oxygen toxicity appears to be most profound in the lung, where exposure is direct [50–52] though genetics, life-style characteristics and environmental factors may profoundly influence mortality and life expectancy [53]. Corroboration and expansion of these findings would be helpful in optimization of medical care and disease management in the geriatric and elderly residents of higher altitudes.

Apparently, pressure seems to have diametrically opposite roles to play in the cause and treatment of cancer. Interestingly, while low pressure has been found to have a preventive effect, high pressure may be effective in the control and treatment of cancer. In the present study, the effect of pressure on the survival and growth of cancer and normal cells was studied at normal atmospheric pressure (1 atmosphere = 14.7 psi) and increased pressures of 18 psi (1.22 atmosphere). Our experimental results have validated a well established theoretical model on cancer cell proliferation rate *in vitro*, however, there is still need for an alternative approach in the form of a prospective experimental physiological study design to be carried out in mice models of breast and lung cancer so that the results may be reproducible in humans in future.

## Materials and methods

### Design of hyperbaric pressure chamber with automatic pressure control system

#### Components

Pressure chamber, pressure sensor (PSI), AVR controller, Wi-Fi module, Auto On/Off relay, Solvonoid valve, LCD display.

#### Software

Online pressure monitoring application.

#### Gas mix

To subject the growing cancer cells to increased pressure, an inert gas (N_2_) was used in addition to the usual 5% CO_2_.The following setups were done to best study the effect of increased pressure on cancer and normal cells (experimentals) *versus* the those maintained at normal pressure (14.7 psi) (controls):

1. Control cancer cells (1x10^5^ cells/mL) were placed in 25 cm^2^ flasks kept in a 5% CO_2_ incubator and maintained at normal atmospheric pressure (14.7 psi) while experimental cancer cells (1x10^5^ cells/mL) were placed in a pressure chamber connected to CO_2_+N_2_ cylinder and maintained at a pressure of 18 psi inside the incubator
2. Control normal cells (1x10^5^ cells/mL) were placed in 25 cm^2^ flasks kept in a 5% CO_2_ incubator and maintained at normal atmospheric pressure (14.7 psi) while experimental normal cells were placed in a pressure chamber connected to CO_2_+N_2_ cylinder and maintained at a pressure of 18 psi inside the incubator

#### Device design

**Fig 1:**
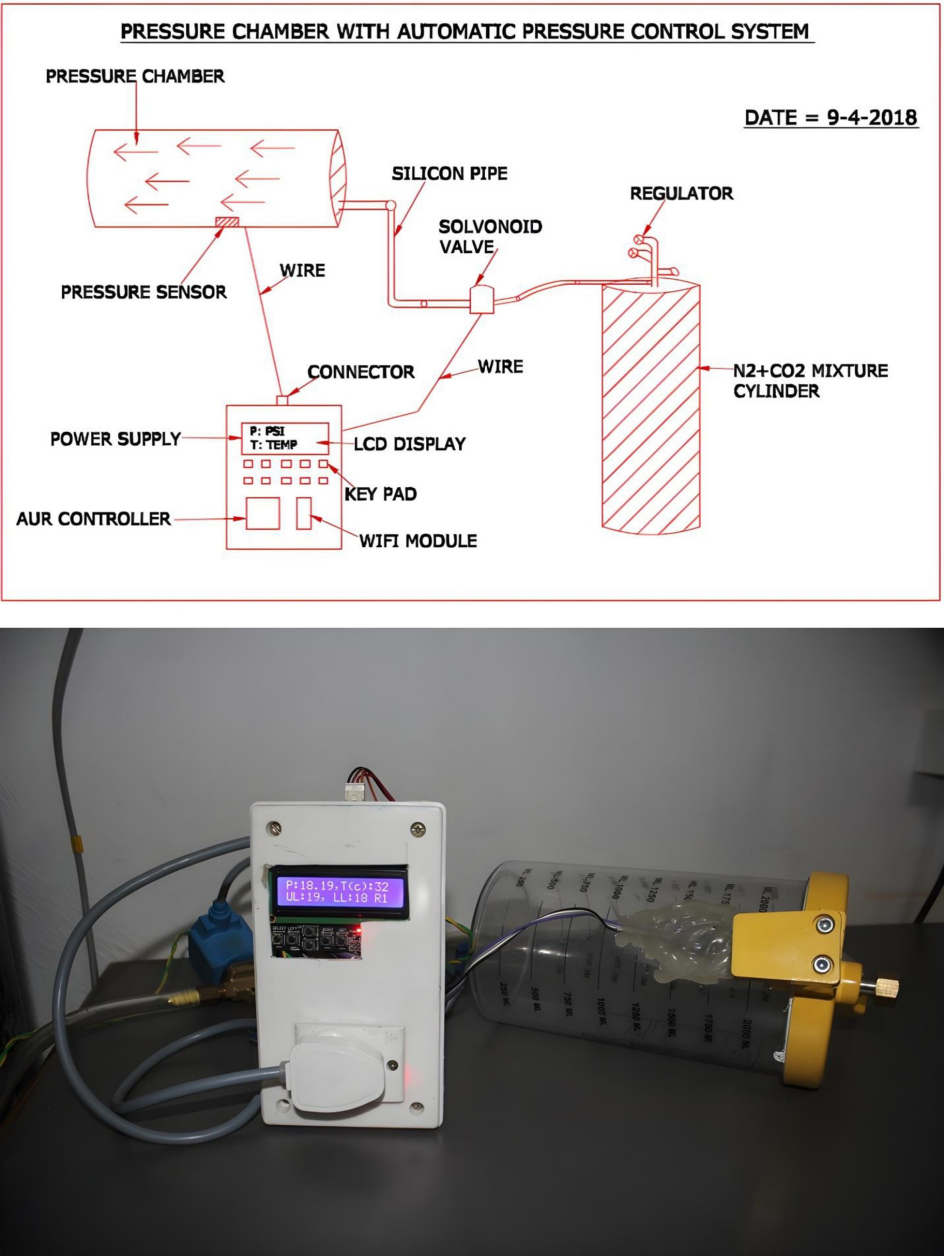
(a). Schematic drawing of the experimental setup. (b). Designed Pressure Chamber.

Hyperbaric Pressure Chamber consists of a pressure sensor which is connected to a control system. Control system has a LCD display, keypad and an AVR controller with enabled data transmission system. The device has the ability to transfer online pressure datasheet 24*7 to a connected mobile or computer system (Fig 1A). Pressure chamber is connected through a flexible silicon pipe to a gas cylinder containing CO_2_+N_2_ mixture. There is a solvonoid valve between cylinder and pressure chamber which is connected to the controller system to maintain the pressure. If the pressure within the chamber falls below 18 psi (set pressure), then the solvonoid valve automatically opens to allow CO_2_+N_2_ gas mixture to pass through it and maintain the desired pressure inside the chamber. After the desired pressure is attained, the solvonoid valve closes automatically till the pressure drops again (Fig 1B).

### Reagents

All chemicals used in cell culture were of analytical grade as reported previously [54, 55].

### Ethical permission

No permission was sought from the Institutional Ethics Committee, Era’s Lucknow Medical College, Lucknow, as the present work did not involve human or animal subjects.

### Cell lines

Three cell lines, namely, MDA-MB-231 (human breast carcinoma), A549 (lung carcinoma), and Vero (normal African green monkey kidney epithelial cells; ATCCCCL-81), obtained from the National Centre for Cell Science, Pune, India, and maintained by serial passaging in Tissue and Cell Culture Lab, Era’s Lucknow Medical College, Era University, Lucknow, were used in the present study.

### Cell culture

For experiments, cells that were trypsinized, seeded and cultured in 25 cm^2^ cell culture flasks (Nest, Tarsons) under conditions of normal pressure (14.7 psi) were designated as controls while those maintained at increased pressure of 18 psi were designated as experimentals as per setups (detailed above) for 48, 72 and 96 h at a pre-chosen density of 1x10^5^ cells/mL for adherence. Results obtained were plotted as cell viability *versus* time period graph based on experiments done in triplicates.

#### Morphological analysis

Observations on cellular morphology were done with the help of 10× and 40× objectives of a phase contrast microscope (Nikon Eclipse Ti, Japan).

#### Cell viability evaluation using Trypan blue dye exclusion assay (TBE)

The assay was done as reported previously [54, 55] to count the number of live and dead cells in control and pressure-treated flasks.

#### Cell cycle analysis by measurement of cellular DNA content

PI dye was used to examine various stages of the cell cycle as well as the DNA content of the cells using flow-cytometry (FACS Lyric, BD Biosciences, USA) as reported previously [56–59]. After the cells were seeded at a population density of 1 x 10^5^ cells/mL for 48h in two different setups, i.e. control cancer cells kept in a 5% CO_2_ incubator and maintained at normal atmospheric pressure (14.7 psi), while experimental cancer cells were placed in a pressure chamber connected to a CO_2_+N_2_ cylinder and maintained at an increased pressure of 18 psi inside the incubator.

#### Annexin V/ PI staining

Further confirmation of the cytostatic and cytotoxic effect of increased pressure on growth and division of cancer and normal cells was done using Tali™ apoptosis AnnexinV/PI staining kit (Molecular Probes, Life Technologies, Invitrogen) following manufacturer’s instructions. Briefly, in separate experiments, MDA-MB-231 and Vero cells were subjected to normal and increased pressure in different setups as detailed above and cultured for 48 h. The cells were thereafter trypsinized, centrifuged, washed and pooled with suspended dead cells. The cells were resuspended in Annexin binding buffer (IX) and stained with Annexin/PI in dark at RT for 20 min. The samples were loaded into Tali^®^ cellular analysis slides and read in a Tali™ image based cytometer. Cell samples were divided into three populations based on the dye labels as apoptotic (green fluorescence), dead (red or yellow fluorescence resulting from a combination of red and green fluorescence), and live (little to no fluorescence).

#### Cell lysis by ultrasonication

MDA-MB-231 cells maintained at normal pressure of 14.7 psi (controls) and increased pressure of 18 psi (experimentals) were suspended in PBS and lysed using a sonicator.

#### Differential centrifugation

Lysates from both control and experimental cells were centrifuged at 1,000 g for 15 min, 10,000 g for 30 min, and subsequently at 100,000 g for 60 min, to obtain nuclear, mitochondrial, microsomal and cytosolic fractions, respectively.

#### Protein estimation

Protein content of the above fractions was determined by Lowry et al. (1951) [60] using BSA as standard.

#### SDS PAGE

Lysates from MDA-MB-231 cells maintained at normal (14.7 psi) and 18 psi pressure were evaluated for their respective protein content by SDS-PAGE 48 h post-treatment. The cell lysates were combined 1:1 with Laemmli sample buffer [61], and this combination was heated for 5 min at 100°C and then placed on ice for 5 min. 20 μL of each sample was electrophoresed through a 10% (w/v) SDS-PAGE gel. Electrophoresis was performed using a Mini-Protean II cell unit (Bio-Rad, Hercules, Calif) as described previously [62]. After electrophoresis, the detection of proteins was done by staining the gel with Coomassie brilliant blue.

#### Scanning electron microscopy (SEM)

SEM of MDA-MB-231 and A549 cells (controls) maintained at normal pressure (14.7 psi) and increased pressure of 18 psi (experimentals) was carried out at Electron Microscopy Unit, Dept. of Pathology, King George’s Medical University, Lucknow. Briefly, both control and experimental cell suspensions were fixed for at least 4 h in a 2.0% (v/v) glutaraldehyde solution in 0.1 mol/L sodium cacodylate buffer. Post-fixation, approximately 1x10^6^ cells were rinsed with PBS thrice for 15 min. The samples were further dehydrated in absolute alcohol and dried for 2.5 h in a Critical point Dryer (Leica EMCPD300) in a CO_2_ medium. The samples were thereby placed on a steel mould. The moulds were then attached to large SEM stubs with double-sided carbon conductive tape and coated with carbon in a SEM spurter machine (Leica EM ACE200). Sample observation was done and microphotographs were taken on a Quanta 250 (FEI) scanning electron microscope.

#### Transmission Electron Microscopy (TEM)

TEM of MDA-MB-231 and A549 cells (controls) maintained at normal pressure (14.7 psi) and increased pressure of 18 psi (experimentals) was also carried out at Electron Microscopy Unit, Dept. of Pathology, King George’s Medical University, Lucknow. Both control and experimental cell suspensions (1.0 mL) were centrifuged at RT and supernatants were discarded. The cell pellets were resuspended and fixed overnight in 4% glutaraldehyde in PBS (pH 7.0) at 4°C. Then, the samples were washed thrice in PBS (pH 7.0) and re-suspended and post-fixed in 1% osmium tetroxide for 1 h at RT. After two washes in PBS and removal of buffer, the cells were resuspended in 2% warm agarose, and the mixture was allowed to solidify on ice. The specimens were cut into pieces not larger than 1 mm^3^ and left in a vial of PBS (pH 7.0) overnight at 4°C. After two washes in distilled water, the specimens were *en bloc* stained in 1% uranyl acetate for 1 min and 15 sec with 0.42% lead citrate. After two washes in distilled water, the samples were dehydrated in graded ethanol at concentrations according to the following schedule: 50% for 45 min, 70% for 45 min, 80% for 50 min, 95% for 1 h and 100% for 1.5 h. Each ethanol concentration was changed thrice during the specified time period. After dehydration, the samples were placed in propylene oxide (98%) for 20 min with two solution changes and infiltrated in 1:1 propylene oxide-Spurr resin overnight under a vacuum (20 inches of Hg). Thereafter, the samples were infiltrated with 100% Spurr resin over 24 h with two solution changes, placed into a Beem capsule with fresh Spurr resin, and put under a vacuum for 4 h. The Spurr-embedded sample was polymerized in a vacuum oven for 8 h at 60°C. The sections were cut at 70 nm on a Reichert Ultracut E ultramicrotome and placed on Formvar-coated 200-mesh copper grids. Grids were stained in 2% aqueous uranyl acetate for 15 min, followed by 0.42% lead citrate (Reynolds) for 5 min. Grids were examined in a Tecnai G^2^ (FEI) transmission electron microscope at 120 kV.

#### Atomic Force Microscopy (AFM)

**Fig 2:**
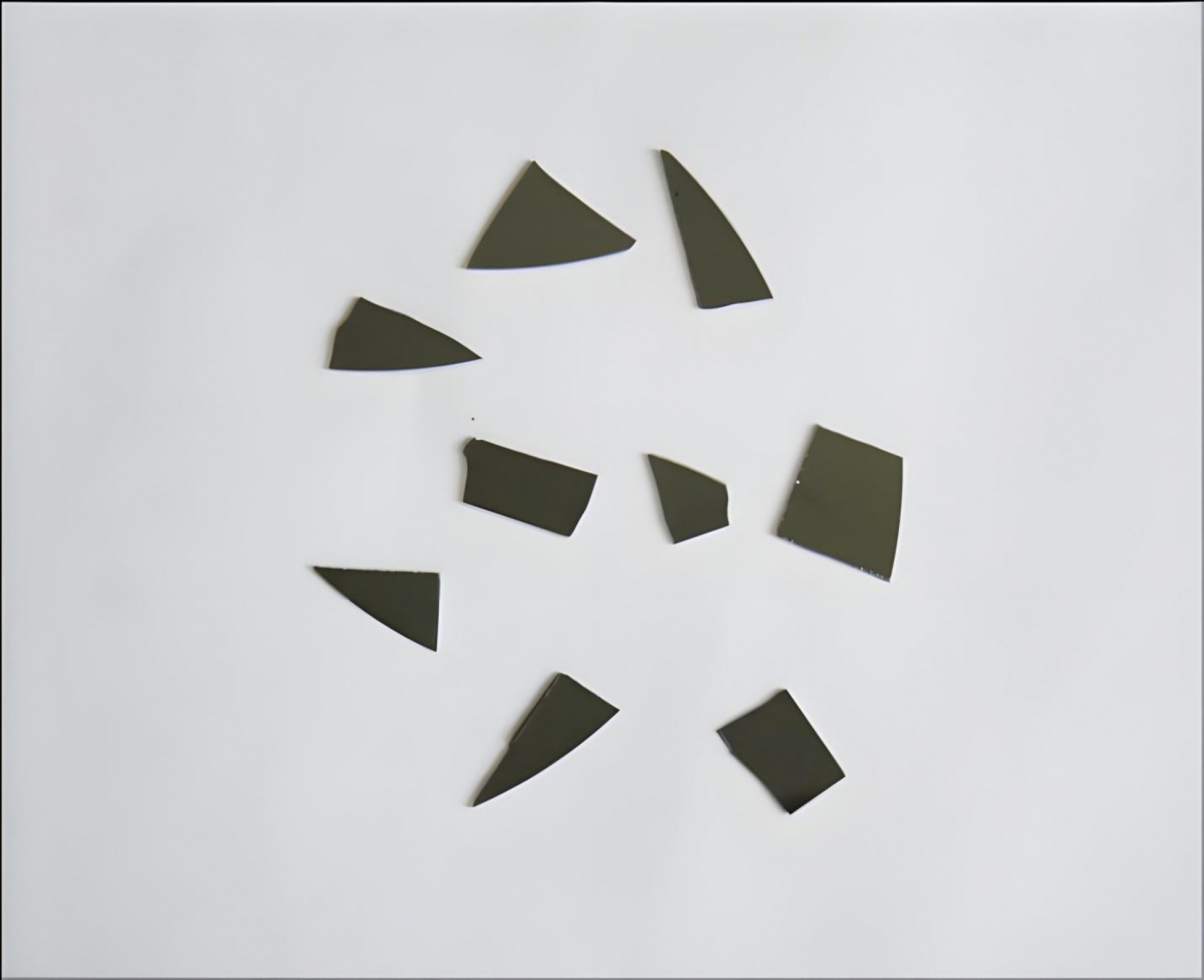
Mica chips for sample immobilization in AFM.

Unprocessed, non-conducting biological materials can be imaged in their native state to atomic resolution using AFM, thus eliminating the need for extensive sample preparation in order to make the sample conducting in order to allow current to pass. This eliminates considerable distortion of sample structure on account of extensive sample processing in case of electron microscopy. AFM of the membrane surface topology of the control and experimental MDA-MB-231 cells was carried at Department of Nanosciences and Nanotechnology, IIT Kanpur, using an Asylum Research MFP 3D Bio AFM (Model AC240BSA-R3) as per the method of Muller and Engel (2007). Since cells are solid entities, the most common substrates used for immobilization of solid samples are mica chips (Whited and Park, 2014) (Fig 2). Briefly, the cells were adhered to mica chips as thin films by applying gentle and even pressure followed by air-drying. The imaging was carried out in high resolution contact mode. The following were the instrument’s technical settings: f(Khz) 70 (50–90), k(N/m) 2(0.6–3.5), scan rate 0.59 Hz, points lines: 256x256, setpoint: 720 mV, imaging mode: AC Mode, Scan size: 10μm, cantilever drive amplitude: 36.58mV, cantilever drive frequency: 68.115 kHz, cantilever spring constant: 1.0 N/m.

### Data interpretation and statistical analysis

Biological data were expressed as the mean ± SD from three independent experiments. Statistical evaluation was determined by oneway ANOVA followed by the Tukey Multiple Comparison Test using GraphPad Prism software (Version 5.1). A p-value of < 0.05 was considered statistically significant.

## Results

**Fig 3:**
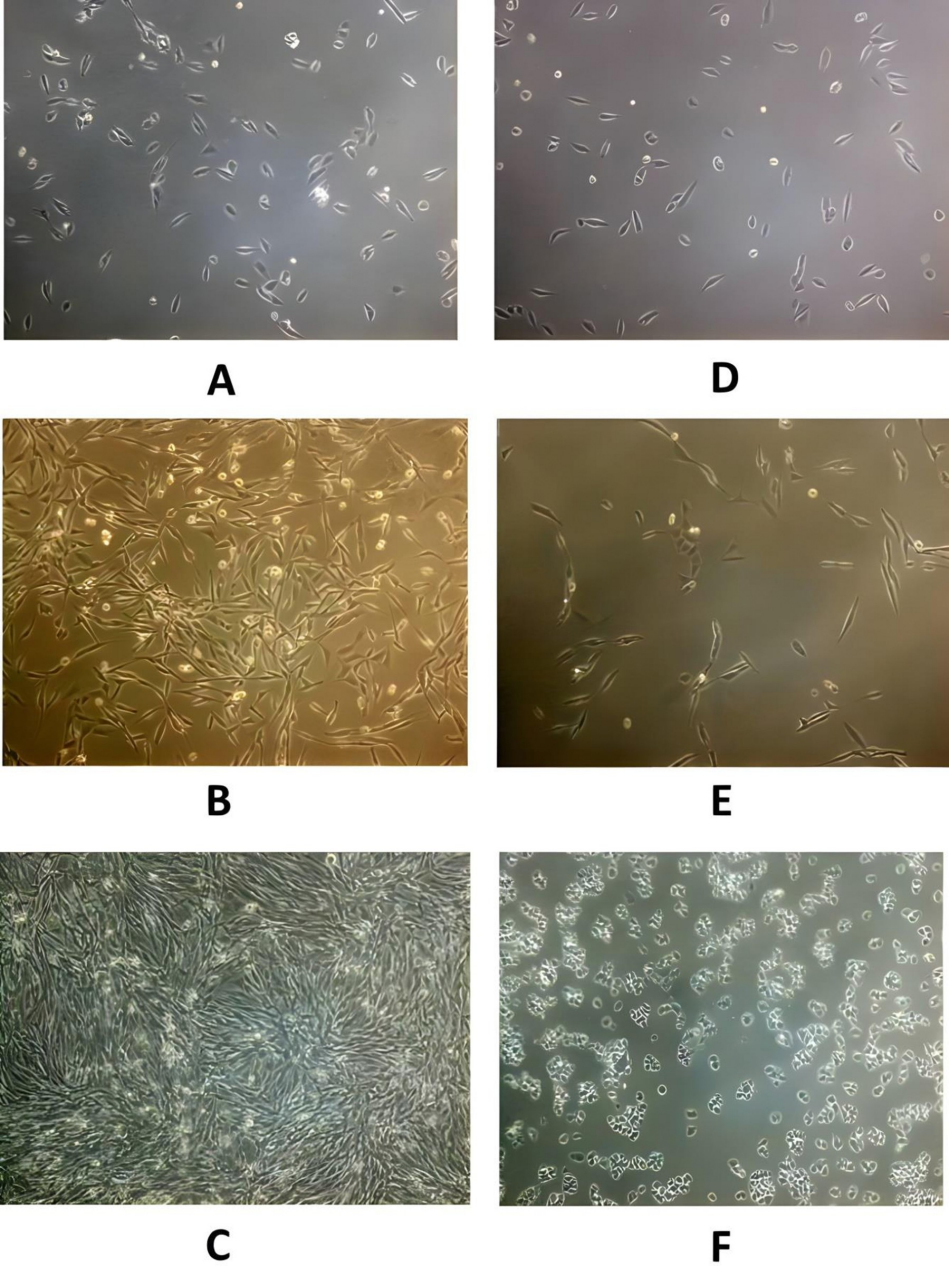
Morphological analysis of control *versus* experimental MDA-MB-231 cells; (A-C) Controls showing MDA-MB-231 human breast cancer cells at 14.7 psi after 24, 48 and 72h respectively (D-F) Cells maintained at 18 psi as per setup 1 described above (Magnification 10x).

**Fig 4:**
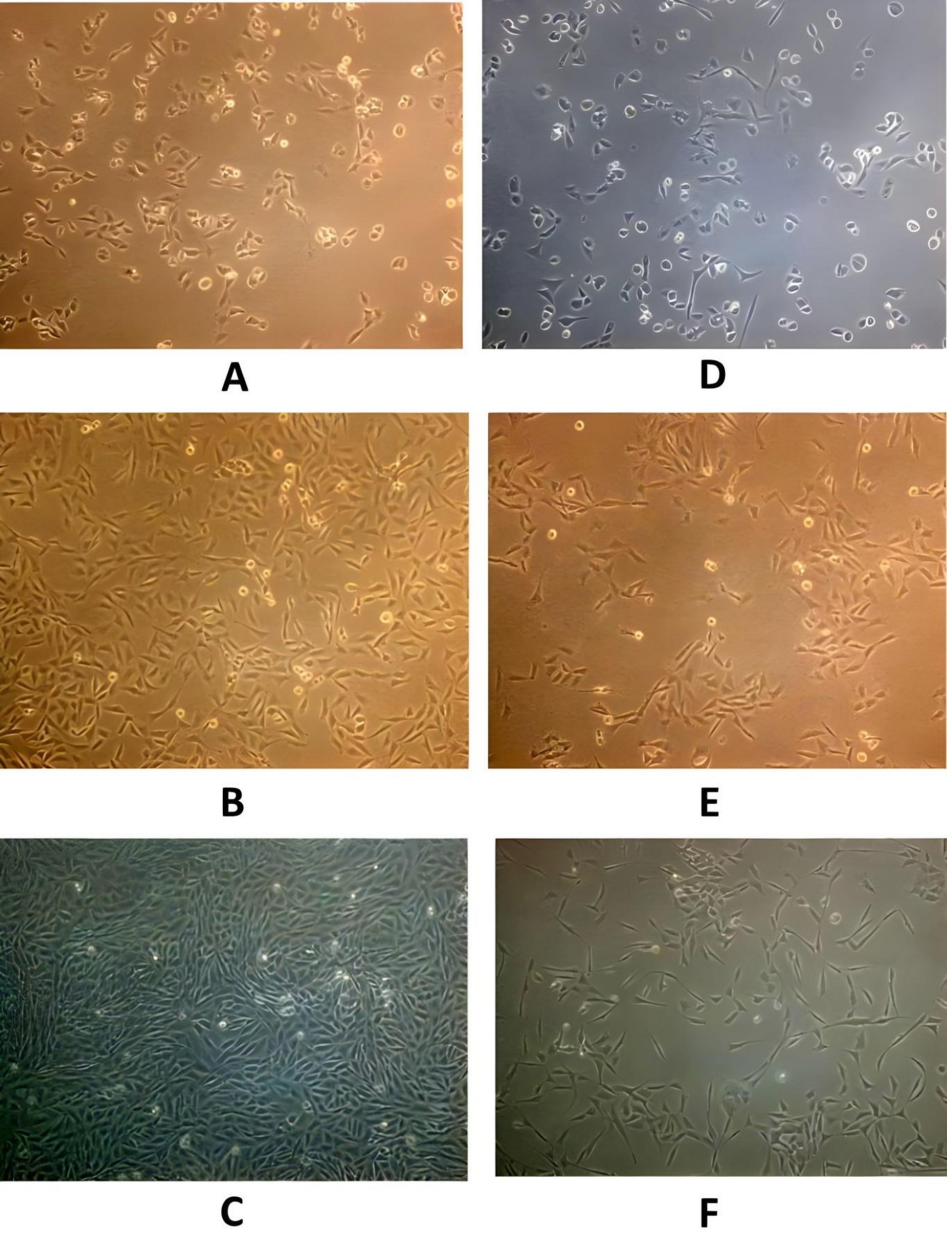
Morphological analysis of control *versus* experimental A549 cells; (A-C) Controls showing A549 human lung cancer cells at 14.7 psi after 24, 48 and 72 h respectively (D-F) Cells maintained at 18 psi as per setup 1 described above (Magnification 10x).

**Fig 5:**
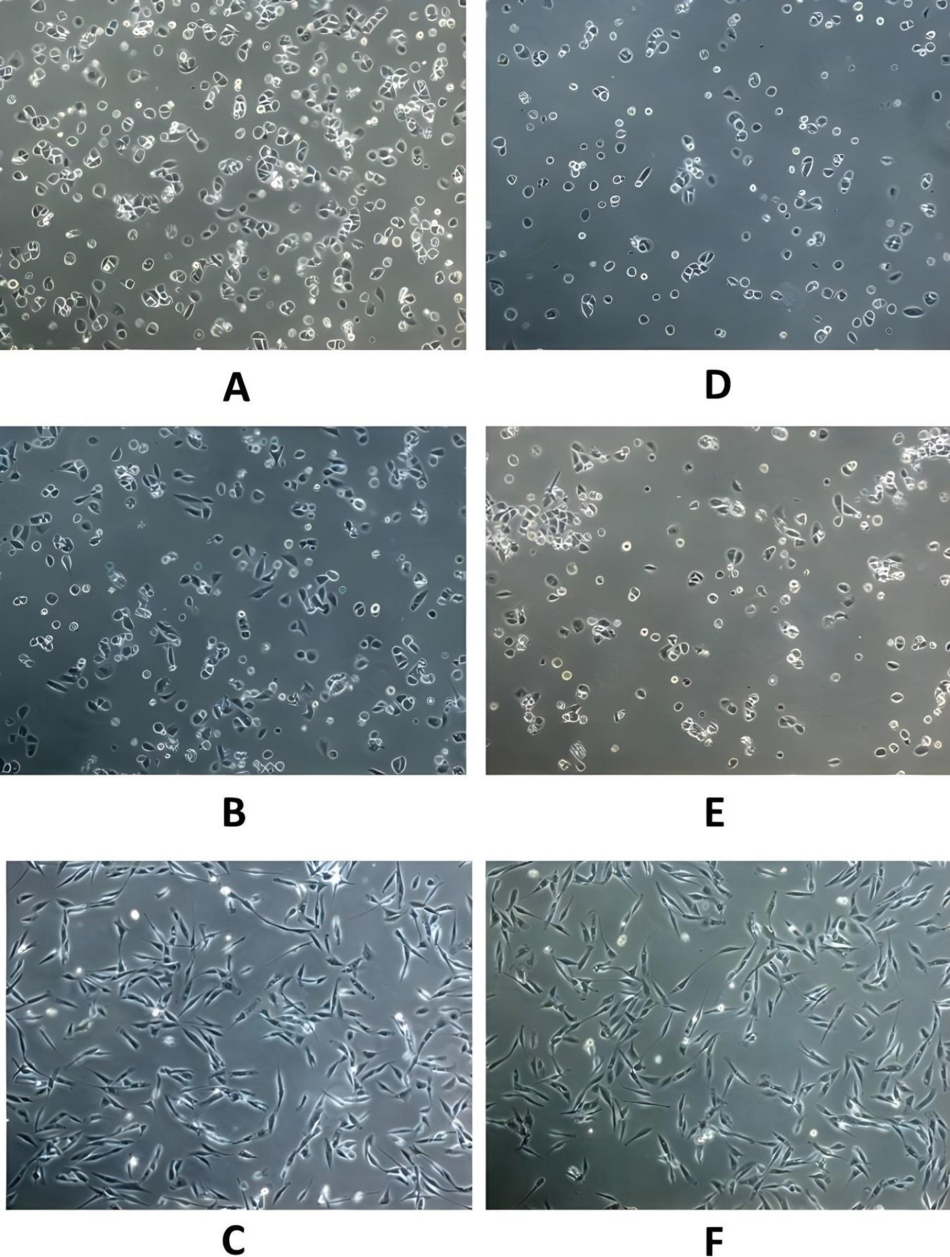
Morphological analysis of control *versus* experimental Vero cells; (A-C) Controls showing Vero normal kidney epithelial cells at 14.7 psi after 24, 48 and 72 h (D-F) Cells maintained at 18 psi as per setup 2 described above (Magnification 10x).

**Fig 6:**
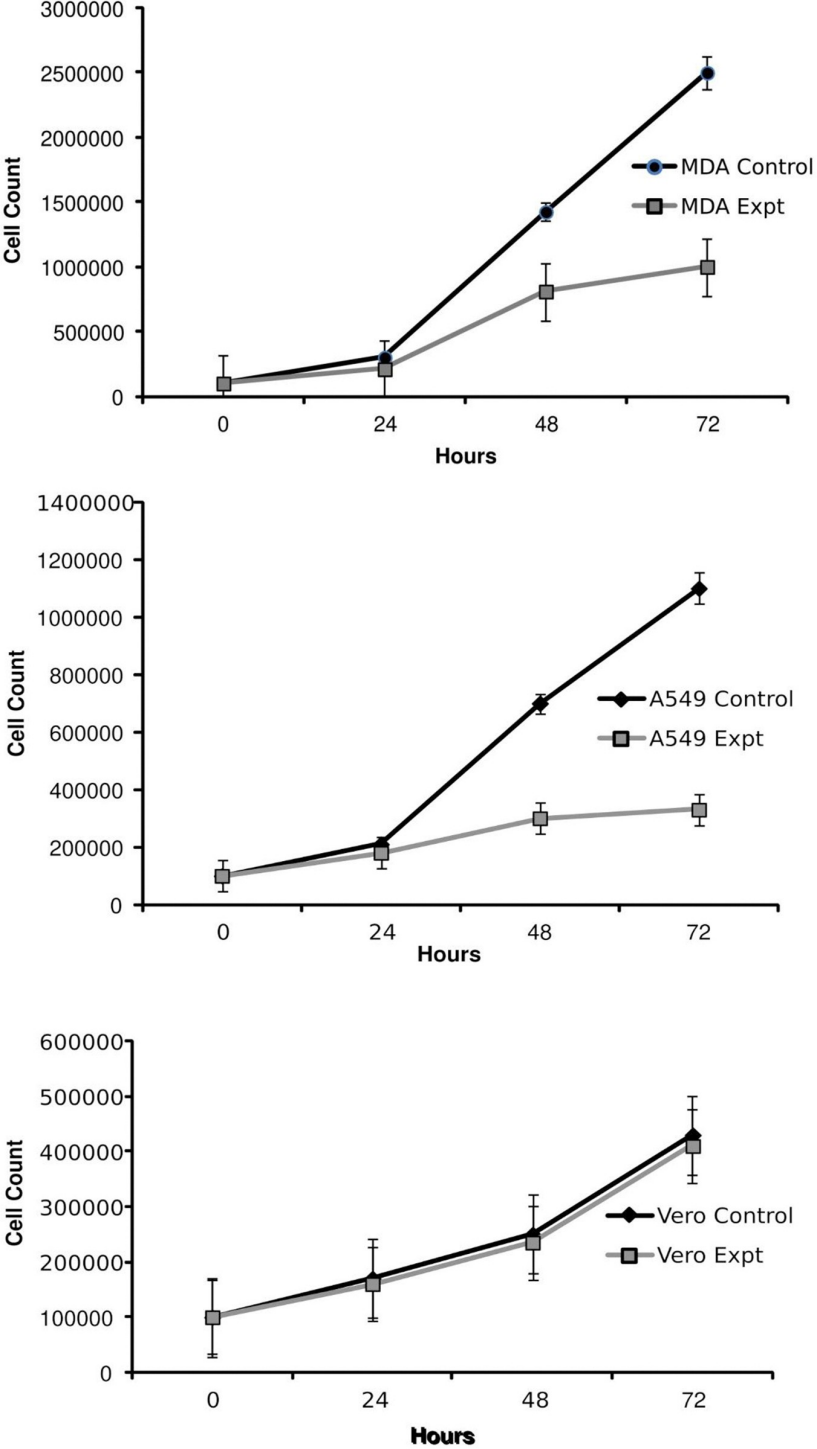
Effect of increased pressure (18 psi) on the growth of (a) MDA-MB-231 (b) A549 and (c) Vero cells (c) *versus* their respective controls at normal pressure (14.7 psi). Results are expressed as mean ± SD of three independent observations.

**Fig 7:**
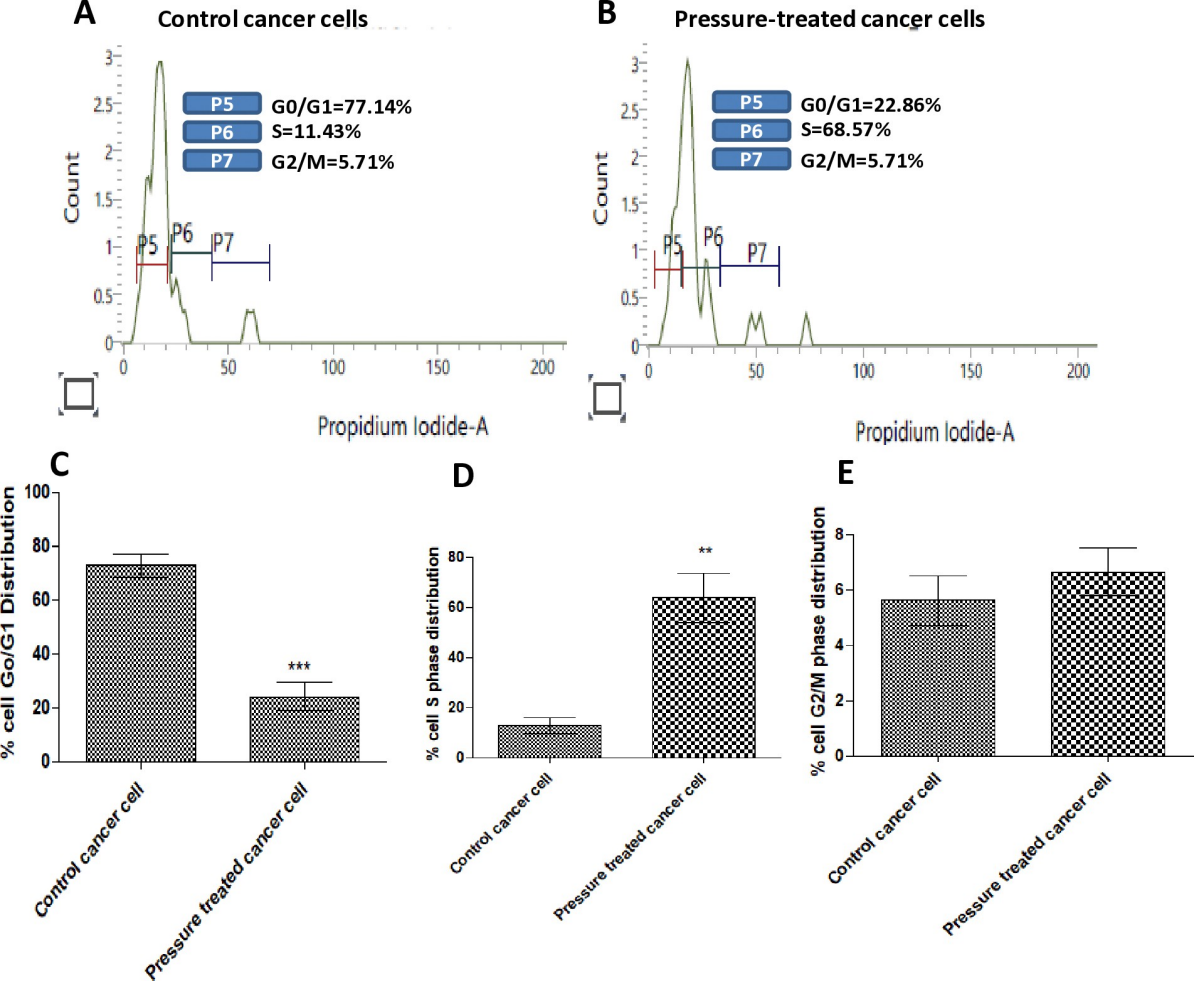
Effect of hyperbaric treatment on cancer cell cycle progression and DNA content by flow-cytometry. Pictorial graph showing mean proportion of cells in different phases of cell cycle (A) Control MDA-MB-231 cells at normal pressure (14.7 psi) and (B) those subjected to 18 psi pressure for 48h; stained with PI and analyzed using flow cytometry. (C-E) The percentage of cell frequency is graphed along the *y-axis*. The data are representative of three independent experiments. Significance of the differences between control cancer cells as compared to pressure treated cancer cells was of *P* value = 0.0002 (***) and *P* value = 0.0010 (**), for G0/G1 and S phase respectively. There was no significance of difference (*P* value = 0.2148) in G2/M phase.

Both cancer cell lines were found to be piezosensitive whereas normal (Vero) cell lines were found to be less sensitive to increased pressure of 18 psi (Figs 3–5, Fig 6C, Table 1). The growth rate was reduced at increased pressure (Figs 3–5 and Fig 6A and 6B); in case of MDA-MB-231 cells, a change in cell morphology was also observed, and the cells presented a more or less rounded appearance as cell division was inhibited (Fig 3F) as compared to the normal elongated spindle-shape of their untreated counterparts. Cell cycle analysis of MDA-MB-231 cells also confirmed that an increase in pressure caused cell cycle arrest in the S phase in pressure-treated MDA-MB-231 cells *versus* those kept at normal pressure (Fig 7A and 7B). As shown in Fig 7, cell population of control cells in S phase was found to be 11.43%, while pressure treatment arrested 68.57% of the cells in S phase. These results imply that S checkpoint of cell cycle is triggered by pressure exposure in MDA-MB-231 cells. The morphologic changes observed under increased pressure conditions suggest that some of the cytoskeletal and/or autolytic proteins were also likely affected. Thus, it is hypothesized that a pressure-mediated inhibition of the cytoskeletal assembly during cell division might contribute to the growth arrest of cancer cells at increased pressure. Inter-subunit interactions within polymeric enzymes, ribosomes, cytoskeletal proteins and signal transduction proteins are thought to be sensitive to increased pressure. Hydrostatic pressure is known to cause the disruption of numerous multimeric proteins because of negative volume changes [63–65]. The effect of high pressure has been studied on the growth of bacterial cells [66, 67]. In our study, it was found that an increase in the surface area of the substratum for the adherent MDA-MB-231 and A549 cells caused a decrease in the pressure-induced cytostatic and cytotoxic effect on cancer cell lines (results not shown).

**Table 1:** Time-dependent evaluation of hyperbaric effect (18psi) on survival of (a) MDA-MB-231, (b) A549 and (c) Vero cells as per setup 1 (cancer cells) and setup 2 (normal cells).

**S. No.**	**Pressure (psi)**	**No. of Live Cells (MDA-MB-231)** *								**% Cytotoxicity** *			
		**Control**			**Treated**								
**1**	18	**24 h**	**48 h**	**72 h**	**24 h**		**48 h**		**72 h**	**24 h**		**48 h**	**72 h**
		3.0x10^5^	1.4x10^6^	2.5x10^6^	2.1x10^5^		8.1x10^5^		1.0x10^6^	20		42.1	60
**S. No.**	**Pressure (psi)**	**No. of Live Cells (A549)** *								**% Cytotoxicity** *			
		**Control**			**Treated**								
**2.**	18	**24 h**	**48 h**	**72 h**	**24 h**	**48 h**		**72 h**		**24 h**	**48 h**		**72 h**
		2.1x10^5^	7.0x10^5^	1.1x10^6^	1.8x10^5^	3.0x10^5^		3.3x10^5^		14.2	57.1		70
**S. No.**	**Pressure (psi)**	**No. of Live Cells (Vero)** *								**% Cytotoxicity** *			
		**Control**			**Treated**								
**3.**	18	**24 h**	**48 h**	**72 h**	**24 h**	**48 h**		**72 h**		**24 h**	**48 h**		**72 h**
		1.7x10^5^	2.5x10^5^	4.3x10^5^	1.6x10^5^	2.4x10^5^		4.1x10^5^		5.9	4		4.7

*Only average values for live cell number and % cytotoxicity of three independent observations for each cell line shown here

**Fig 8:**
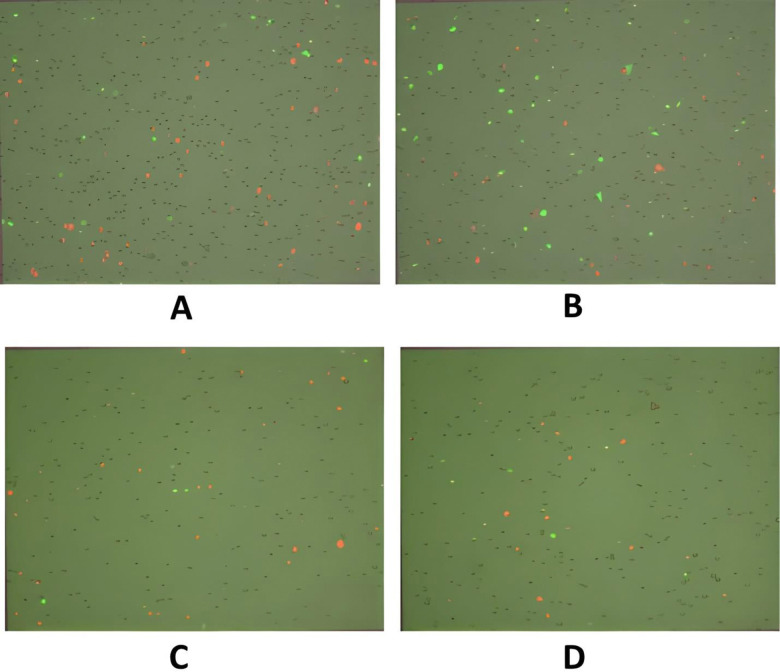
Apoptosis assay using Annexin V/PI to determine cell viability and health. (a) MDA-MB-231 cells at 14.7 psi (controls) and (b) MDA-MB-231 cells maintained at 18 psi (experimentals) for 48 h (c) Vero cells at 14.7 psi (controls) and (d) Vero cells maintained at 18 psi (experimentals) for 48 h. Green fluorescent cells were labeled with Annexin and designated apoptotic; PI-labeled cells fluoresced red and were dead; yellow fluorescent cells were labeled with both Annexin and PI and were also counted as dead; whereas unstained cells were counted as live.

**Fig 9:**
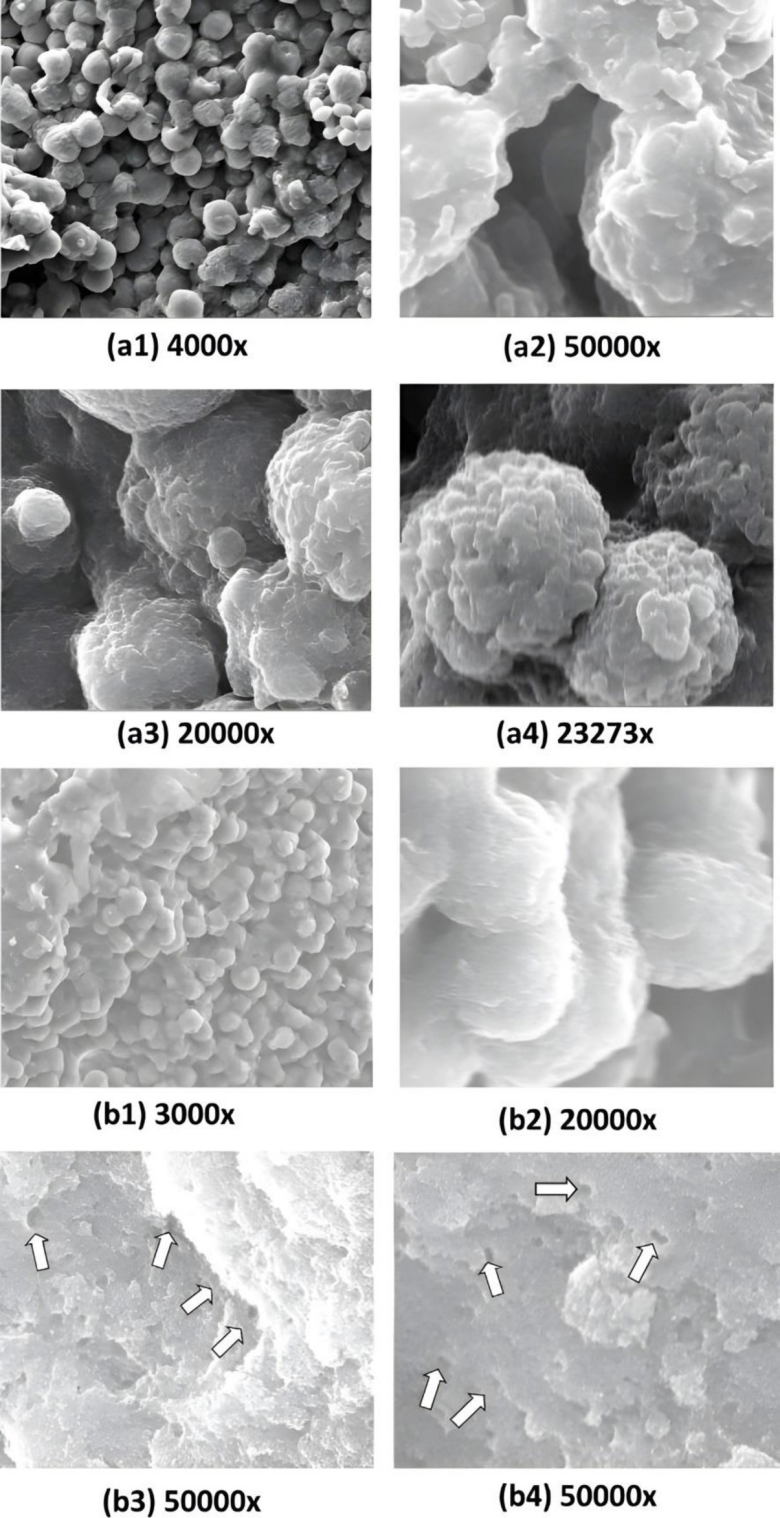
Effects of hyperbaric treatment on MDA-MB-231 cells. Scanning electron micrographs showing MDA-MB-231 cells at 14.7 psi (a1-a4) and 18 psi pressure (b1-b4) at various magnifications. (a1) large number of cells seen (a2) cell membranes appeared well-defined and mostly regular; no lamellipodia or invadopodia could be made out at this magnification; no spikes or tentacles seen (a3, a4) normal looking multiple cells seen (b1, b2) cells appeared almost similar as in controls (a1, a2) because of unappreciated differences (b3) surface of cells appeared granular at this magnification; cell membranes showed pore-like structures, no other details could be made out at this magnification (b4) cell surface from a different area; pore-like structures were more prominent; granular congregations were more apparently defined (a1, a2: 30 and 2 μm bars; a3 and a4: 5 μm bars b1, b2: 30 and 4 μm bars; b3 and b4: 4 μm bars).

Annexin/PI staining of pressure-treated MDA-MB-231 cells did not reveal a significant number of cells undergoing apoptosis *versus* the matched untreated controls (Fig 8A and 8B, Table 2A). Annexin/PI analysis revealed no significant cytotoxic effect of increased pressure, rather it exerted a remarkable cytostatic effect on both type of cancer cells. Treated Vero cells, on the other hand did not show any significant change in number as compared to their matched controls maintained at normal atmospheric pressure (Fig 8C and 8D, Table 2B). The absence of significant number of cells undergoing apoptosis at 18 psi (Fig 6) suggests that increased pressure causes cellular injury by damaging cell membranes thus, inducing necrotic cell death. Damage to the cell membranes was evident in SEM micrographs of control *versus* pressure-treated MDA-MB-231 and A549 cells in the form of characteristic ‘pores’ and ‘pits’ on the surface of pressure-treated MDA-MB-231 and A549 cells, respectively (Figs 9 (b3), (b4) and 10 (b1-b4)). The cells also presented a more contracted appearance as compared to their matched controls maintained at normal atmospheric pressure. It has been found that the cell membrane is a sensitive target and mediator of pressure induced stress response and lethal effects [68]. TEM analysis also revealed remarkable intracellular differences between pressure-treated and untreated MDA-MB-231 and A549 cells (Figs 11 and 12). Pressure-treated MDA-MB-231 and A549 cells displayed nuclei with condensed and disrupted chromatin, irregular thickenings and abnormalities in structure of nuclear membranes and poorly defined cellular vesicles as compared to their untreated counterparts (Figs 11 (b1-b4) and 12 (b1, b2)). AFM analysis of control *versus* pressure-treated MDA-MB-231 cells also provided further confirmation to SEM results that increased pressure causes damage to cell membrane leading to necrosis (Fig 13). Cell membranes of intact pressure treated cells presented a distinctly granular appearance with characteristic pore-like structures (Fig 13b) that were more prominent in the microsomal fractions of the same (Fig 13C). Overall, the effects of pressure were most apparent at the cell surface. This was found to be in agreement with our reasoning that the effects of pressure are most prominent at the site of application and decrease with distance from point of application. There was increase in protein-content of various fractions of cell lysates derived from MDA-MB-231 cells maintained at 18 psi pressure *vis-à-vis* those maintained at normal (14.7) psi as evident from S1 Table. SDS-PAGE analysis revealed an increase in protein expression in all differentially centrifugated fractions of MDA-MB-231 cells maintained at 18 psi due to high pressure-induced cellular stress response (S1 Fig).

**Fig 10:**
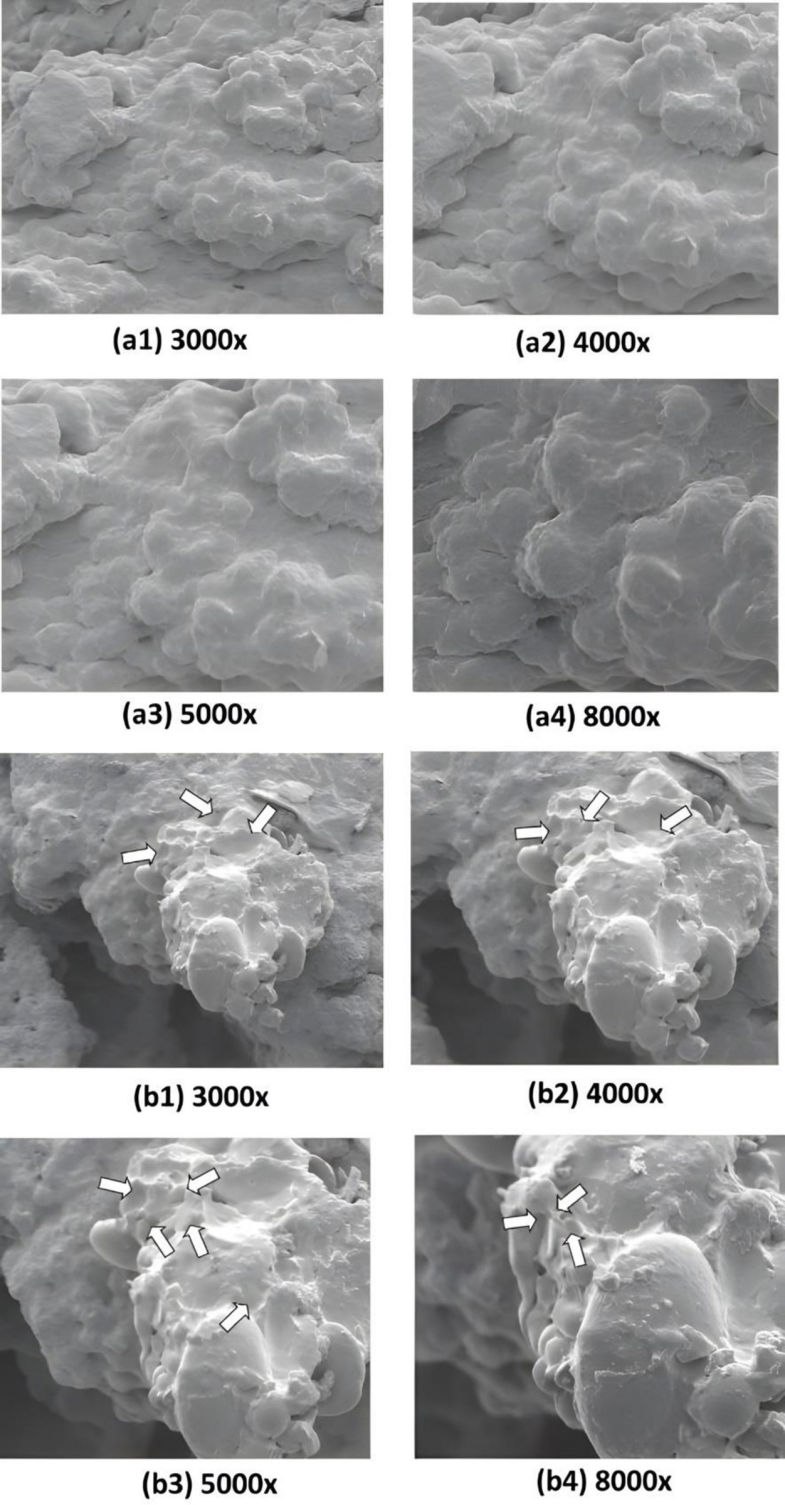
Effects of hyperbaric treatment (18psi) on A549 cells. Scanning electron micrographs showing A549 cells at 14.7 psi (a1-a4) and 18 psi pressure (b1-b4) at various magnifications. (a1-a4) Membrane surface showed irregularities; no lamellipodia or invadopodia could be made out at this magnification (b1-b3) Cells in cluster; although outer membrane appeared irregular in a majority of cells, the surface showed several pits or depression like areas which were irregular and not communicating with each other; no other structural details of surface membrane like ruffling, etc., could be made out; no lamellipodia or invadopodia could be seen (b4) very sharp pits and well-defined membranes could be seen at this magnification; smaller pits well-defined as compared to larges ones (a1, a2: 40 and 30 μm bars; a3 and a4: 20 and 10μm bars; b1, b2: 40 and 30 μm bars; b3 and b4: 20 and 10 μm bars).

**Fig 11:**
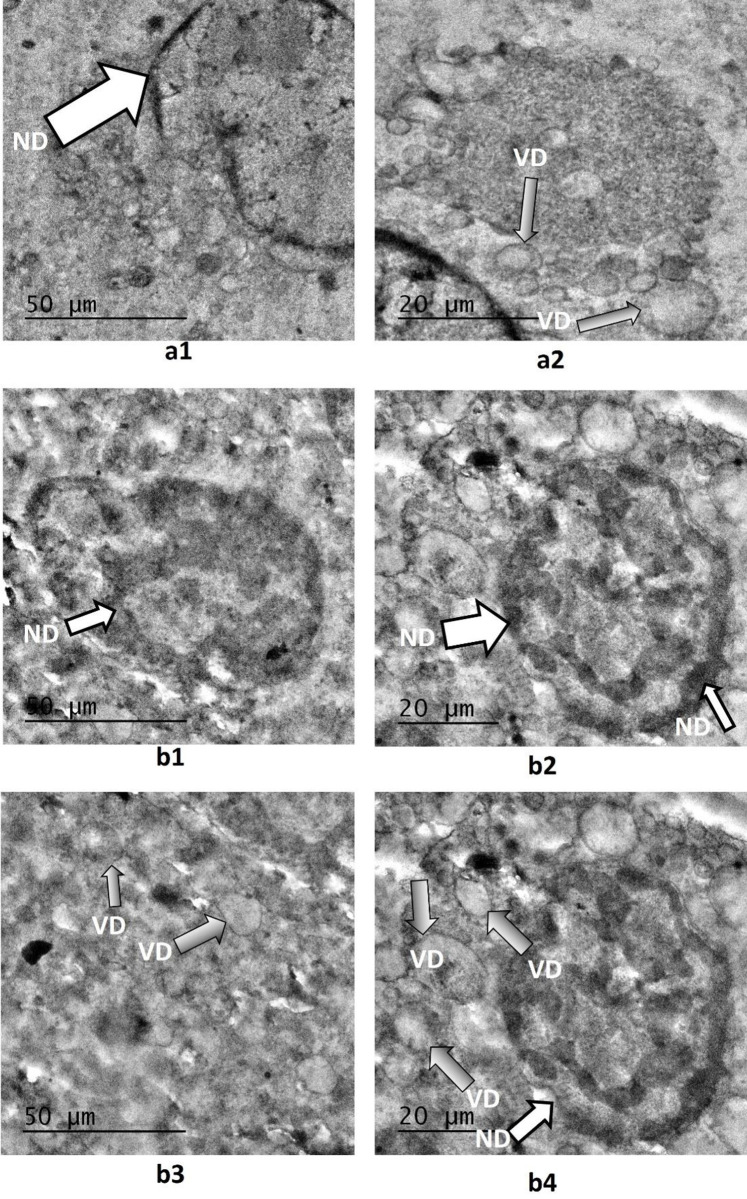
Effects of hyperbaric treatment on MDA cells. (a1, a2) TEM images of untreated MDA controls showed almost normal morphology; the nuclear membrane showed mild chromatin thickening and at one place there appeared a membrane discontinuity (a1); liposomes and other vesicles appeared normal (a2) (b1-b4) TEM images of pressure-treated MDA cells displayed thickened and disrupted nuclear membrane with condensed chromatin (b1, b2), no well-defined cellular vesicles were seen (b3, b4) (ND: Nuclear details, VD: Vesicular details).

**Fig 12:**
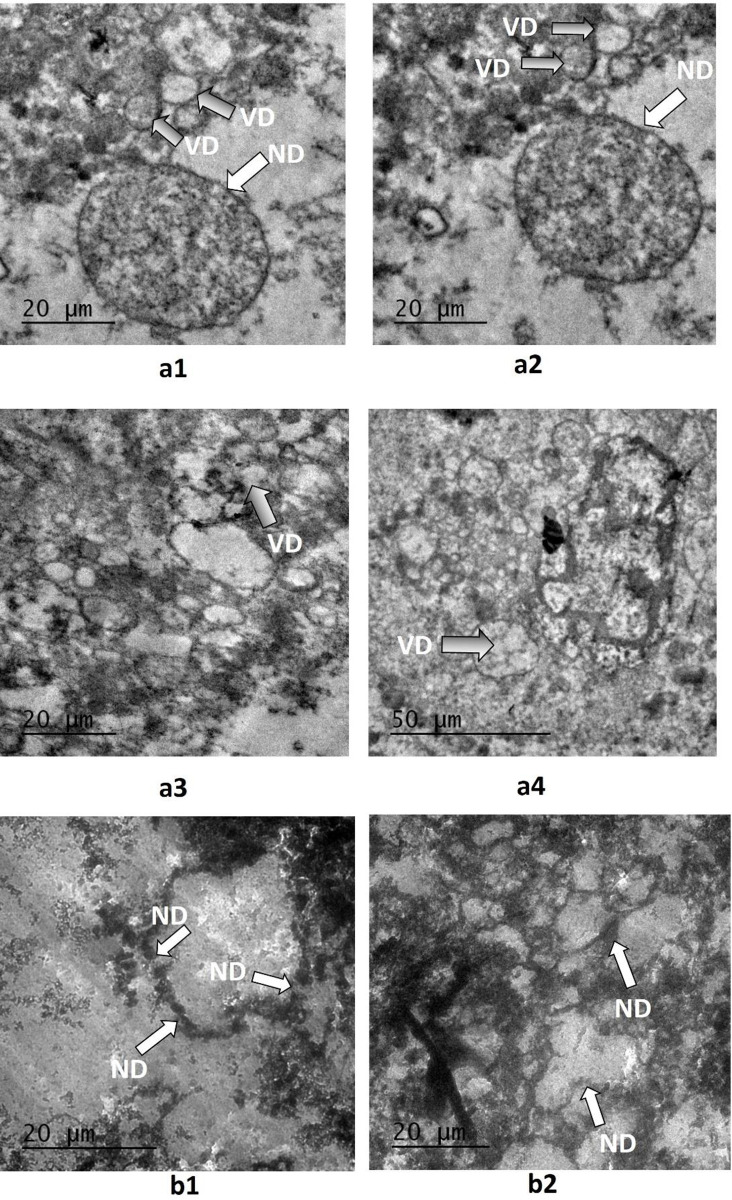
Effects of hyperbaric treatment on A549 cells. (a1-a4) TEM images of untreated A549 control cells displayed very well-defined cell nuclei with intact and continuous membranes; vesicles were numerous and well-defined (b1, b2) TEM images of pressure-treated A549 cells showed nuclear pores and nuclei with abnormal morphology with prominent leakage of nuclear proteins which needs further biochemical assessment. Chromatin appeared disrupted and condensed and nuclear membrane showed irregular thickening. No mitochondria, microvilli, vesicles or Golgi apparatus could be seen in the pictures (ND: Nuclear details, VD: Vesicular details).

**Fig 13:**
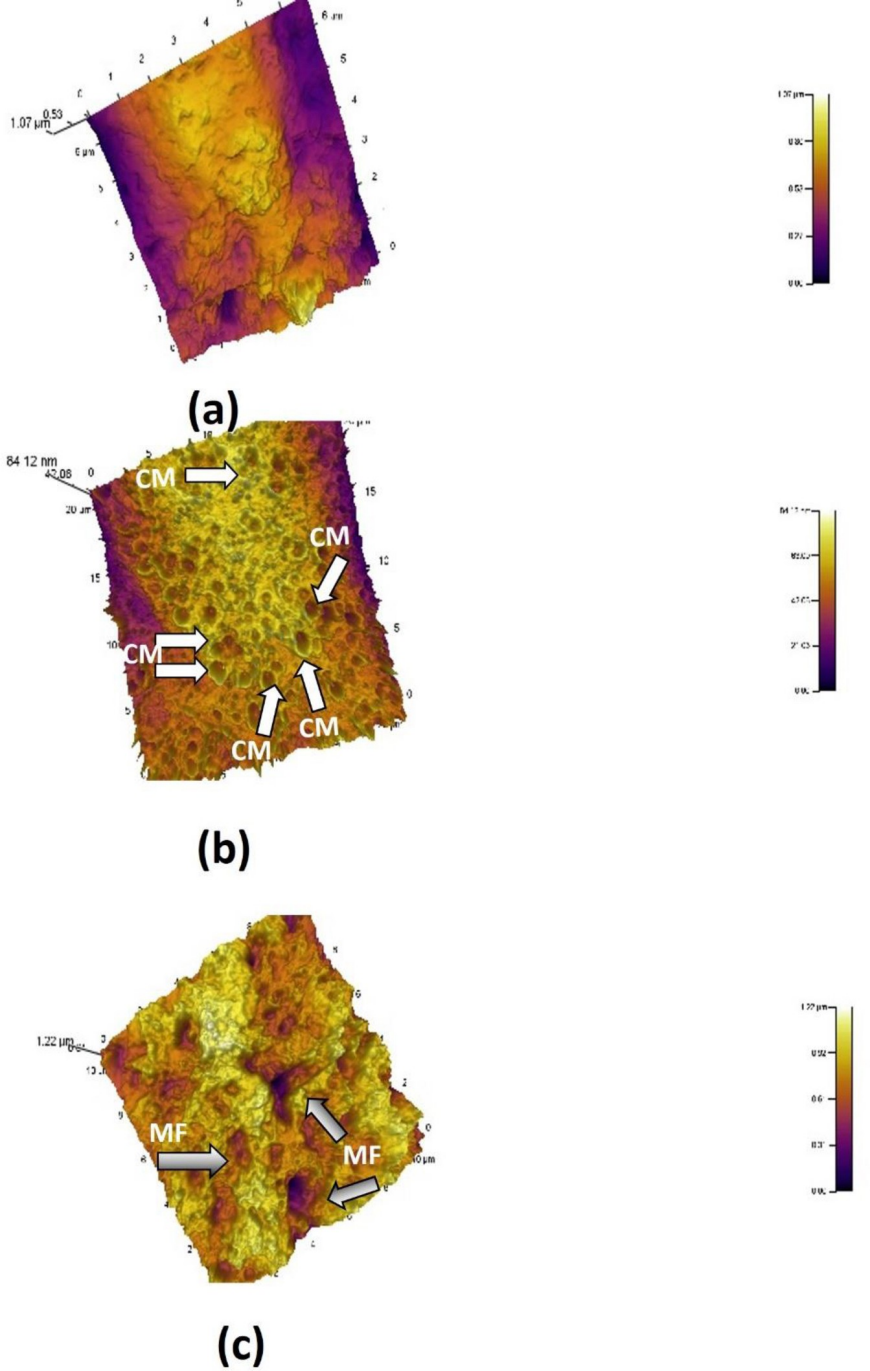
High-resolution AFM of membrane of (a) untreated (b) pressure-treated MDA cells; and (c) microsomal fraction of pressure-treated MDA cells. (a) cell membranes appeared well-defined and mostly regular except for a few irregularities (b) cell surface appeared to be granular and cell membranes showed characteristic pore-like structures (c) cell surface appeared more irregular and granular and more were more pronounced and conspicuous. Contact mode AFM topographs were recorded at scan rate 0.59 Hz, scan size: 10μm, cantilever spring constant: 1.0 N/m, as mentioned din materials and methods (Whole cell membrane details: CM, Microsomal fraction details: MF).

**Table 2:** Live, Dead and Apoptotic populations in Apoptosis assay of high pressure-treated (a) MDA-MB-231 (b) A549 and (c) Vero cells after 48 h.

**(a)**																
**S. No.**	**Ctrl/Expt MDA-MB-231 cells**		**Total Cell No. (cells/mL)**		**Live (cells/mL)**		**%**		**Dead (cells/mL)**		**%**		**Apoptotic (cells/mL)**		**%**	
1.	Control		1.40x10^6^		1.29x10^6^		92.1		8.1x10^4^		5.8		3x10^4^		2	
2.	Experimental*		8.1x10^5^		4.7x10^5^		58		3.2x10^5^		39		2.4x10^4^		3	
**(b)**																
**S. No.**		**Ctrl/Expt Vero Cells**		**Total Cell No. (cells/mL)**		**Live (cells/mL)**		**%**		**Dead (cells/mL)**		**%**		**Apoptotic (cells/mL)**		**%**
1.		Control		2.03x10^5^		1.93x10^5^		95		0.71x10^4^		4		2.0x10^3^		1
2.		Experimental *		2.46x10^5^		2.26x10^5^		92		1.5x10^4^		6		5.0x10^3^		2

Additionally, several pilot experiments were carried out on 15–17 psi before selection of 18psi as the experimental value for pressure. Results obtained at 15–17 psi were either non-significant or did not show any difference in the growth and morphology of cancer cells *vis-à-vis* those maintained at normal atmospheric pressure of 14.7 psi. The authors have included the results of pilot studies on MDA-MB-231 and A549 cells carried out at 16psi as S2 Table.

## Discussion

Pressure leads to decrease in the volume of the system in case of liquids. Pressure leads to a repression of biochemical phenomena leading to positive volume changes. In case of solid phase e.g. cells, high pressure has been demonstrated to exert a growth-inhibitory and cytostatic effect on actively dividing cells [67, 69]. Our study demonstrated that a pressure of 18 psi had cytotoxic as well as cytostatic effects on treated cancer cells (Figs 3, 4, 7, Tables 1 and 2).

High pressure is known to induce unique stress responses in mesophilic bacteria, such as expression of specific proteins, osmolyte accumulation, and metabolic changes [63, 70–75]. Molina-Höppner et al (2003) have shown that pressure has the ability to alter the morphology and functionality of cytoskeleton proteins of lactic acid bacteria [67]. The response of lactic acid bacteria to high pressure treatment has shown alteration in cell organization, proteome expression as well as changes in the integrity of the cell membrane [68]. Pressure stress affects all aspects of cellular physiology including membrane physiology, transport characteristics, metabolism, transcription and translation. Pressure has been found to induce various responses in cells such as those being pressure-inducible, -repressible and–independent [76]. Our study done in the context of cancer and normal cells demonstrated that increase in pressure alters cellular morphology and physiology and compromises the integrity of the cell membrane.

Stress response of cells to high pressure treatment can be gauged by an analysis of their proteomes [67] that can be used to detect the presence or absence of soluble proteins before and after pressure treatment. In the lactic acid bacterium *L*. *sanfranciscensis*, a number of proteins have been found to be induced after growth at a pressure of 80 MPa for 30 min. This high pressure treatment has been found to reduce the growth rate by this bacterial species by 90%. Several proteins have been found to be induced in pressure *versus* untreated cells by 2D- electrophoresis; the main ones being alcohol dehydrogenase and the heat shock protein 60 known as groEL [67]. In the present study, SDS-Page analysis of subcellular fractions from experimental MDA-MB-231 cells maintained at 18 psi showed an increase in protein expression as well as presence of new bands *vis-à-vis* control cells at 14.7 psi (S1 Fig).

Increase in pressure can also cause irreversible damage to ion channels. Ion channel activation and inactivation occur due to changes in conformational rearrangements of channel proteins as a result of volume changes [77]. These volume changes are highly susceptible to changes in ambient pressure. Depending on the pressure level, channel function may be irreversibly altered by pressure. An altered/disturbed channel function may cause irreversible damage to the cell leading to its premature death [77] due to disturbance in ion homeostasis inside and outside the cell. Interestingly, certain pressure gated-channels also known as mechanosensitive (MS) or stretch-gated ion channels are known to occur in cardiac tissue. These MS channels, which use external mechanical force (pressure) to open and allow passage of ions through them [78] are sensitive to even slight changes in pressure in the surrounding tissue.

The tolerance of mammalian cells to increased pressure is less than those of microbial cells. Very few studies have been done on mammalian cells in context of effects of pressure on membrane physiology and function. The maximum pressure that cells from higher organisms can tolerate without major effects on membrane dynamics and ion channel function typically ranges between 1450.4 and approx 8702.3 psi and also the cell type [79–85]. However, many eukaryotic cells have a definite limit up to which they can resist cellular alterations [83, 86–88]. Intracellularly, protein misfolding and unfolding, protein denaturation and dissociation usually occur in the 14503.8–43511.3 psi range [65, 89–91]. As can be expected, cancer cells, due to their disturbed physiology will be more sensitive to even small increments of pressure as compared to their normal counterparts. This was found to be the case in the present study. A few microorganisms can withstand long-term exposures to extremely high pressures [92]. This means that there is a difference in the membrane composition and hence, membrane properties of microorganisms as compared to their mammalian counterparts. The present study thus, can prove to be a valuable tool in future for evaluation of mechanisms underlying cellular high-pressure tolerance [65, 82, 93–98].

Pande et al. (2012) have recently reported that HBOT caused a delay in tumor growth while an acceleration in tumor growth occurs post-HBOT cessation. On the other hand, it has been found that mice which underwent HBOT died prematurely than those that did not undergo HBOT [5]. The said study involved a daily exposure of 2 h of oxygen at 16 and 17.4 psi for 21 days in a hyperbaric pressure chamber [5]. Our study differed from that of Pande et al., 2012 in that it was carried out *in vitro* on cancer cell lines rather than on animal models. Secondly, to provide hyperbaric conditions to the dividing cancer cells; N_2_ instead of O_2_ was used in addition to the normal 5% CO_2_ for producing HCO_3_ buffering capacity. By using N_2_ for creating hyperbaric conditions, our results were found to be consistent in causing lesser cytotoxicity in cancer cells due to decreased generation of free oxygen radicals. Thus, hyperbaric nitrogen therapy may exert its effect on the tumor microenvironment through regulation of cellular metabolism (increased protein synthesis) or tumor microenvironment (decreased cell proliferation).

To conclude, the present study, details the effect of applying increased pressure (18 psi) through a specially designed device on the survival and viability of breast cancer MDA-MB-231 and lung cancer A549 cells *versus* breast and lung cancer cells at normal atmospheric pressure as well as normal (Vero) cells treated in similar manner as above. SEM and AFM of intact MDA-MB-231 cells and microsomal fractions of these cells was also performed to ascertain any conformational changes in the membrane structure of cells subjected to increased pressure *vis-à-vis* those at normal atmospheric pressure. In future, our aim would be to investigate in detail the effects of increased pressure on membrane physiology and function of breast and lung cancer cells and to ascertain any changes in the conformation of the membrane-bound proteins of cancer cells subjected to increased pressure.

## Conclusion

Promising results against breast and lung cancer cell lines are paving the way for development of a non-invasive treatment modality for cancer that will selectively target and destroy both metastatic and non-metastatic cancer cells in humans, without affecting the normal cells, a serious drawback of chemo and radiotherapy. It is planned to test the strategy in future on hospital-based population and rural-community programs. The study is aimed at ameliorating the pain and bringing relief to millions of people in this part of the world who are either suffering from this deadly and debilitating disease or are in its early stages.

## Supporting information

S1 Table Protein content of fractionated cell lysates. (DOCX)

S2 Table Time-dependent evaluation of hyperbaric effect (16psi) on survival of MDA-MB-231 and A549 cells. *Only average values for live cell number and % cytotoxicity of three independent observations for each cell line shown here.
(DOCX)

S1 Fig SDS PAGE of fractionated cell lysates of MDA-MB-231 cells. Lane 1: nuclear (0.65μg) 2: mitochondrial (0.08 μg) 3: microsomal (0.06 μg), 4,5: cytosolic (0.15 μg) fractions from MDA-MB-231 cells maintained at 14.7 psi; Lane 6: nuclear (1.5 μg) 7: mitochondrial (0.62 μg) 8: microsomal (0.45 μg), 9,10: cytosolic (0.50 μg) fractions from MDA-MB-231 cells maintained at 18 psi.
(DOCX)

S2 Fig MDA-MB-231. (DOCX)

S3 Fig A549. (DOCX)
