# Supplementary material for: Effects of pressure on the survival and viability of cancer cells in vitro: An analytical study
Source: PLoS One. 2025 Feb 25;20(2):e0311685. doi: 10.1371/journal.pone.0311685 (PMC11856587; doi:10.1371/journal.pone.0311685)
Supplement: S1 Table — (DOCX) [file pone.0311685.s001.docx]

**Suppl. Table 1. Protein content of fractionated cell lysates**

| **S. No.** | **Fraction** | **MDA-MB-231 maintained at 14.7 psi**  **(Protein in µg/mL)** | **MDA-MB-231 maintained at 14.7 psi**  **(Protein in µg/mL)** |
| --- | --- | --- | --- |
| **1.** | **Nuclear** | **32.5±2.5** | **75±2.8** |
| **2.** | **Mitochondrial** | **4.0±2.8** | **31±3.1** |
| **3.** | **Microsomal** | **3.0±1.2** | **22.5±2.2** |
| **4.** | **Cytosolic** | **7.5±5.4** | **25±5.6** |
