# Supplementary material for: Effects of pressure on the survival and viability of cancer cells in vitro: An analytical study
Source: PLoS One. 2025 Feb 25;20(2):e0311685. doi: 10.1371/journal.pone.0311685 (PMC11856587; doi:10.1371/journal.pone.0311685)
Supplement: S2 Table — *Only average values for live cell number and % cytotoxicity of three independent observations for each cell line shown here. (DOCX) [file pone.0311685.s002.docx]

**Suppl. Table 2.** Time-dependent evaluation of hyperbaric effect (16psi) on survival of MDA-MB-231 and A549 cells.

***Only average values for live cell number and % cytotoxicity of three independent observations for each cell line shown here**

| **S. No.** | **Pressure (psi)** | **No. of Live Cells (MDA-MB-231)*** | | | | | | **% Cytotoxicity*** | | |
| --- | --- | --- | --- | --- | --- | --- | --- | --- | --- | --- |
|  |  | **Control** | | | **Treated** | | |  |  |  |
| **1.** | 16 | **24 h** | **48 h** | **72 h** | **24 h** | **48 h** | **72 h** | **24 h** | **48 h** | **72 h** |
|  |  | 1.39x10^6^ | 2.17x10^6^ | 2.41x10^6^ | 1.28x10^6^ | 1.97x10^6^ | 1.86x10^6^ | 7.9 | 9.2 | 22.8 |
| **S. No.** | **Pressure (psi)** | **No. of Live Cells (A549)*** | | | | | | **% Cytotoxicity*** | | |
|  |  | **Control** | | | **Treated** | | |  |  |  |
| **2.** | 16 | **24 h** | **48 h** | **72 h** | **24 h** | **48 h** | **72 h** | **24h** | **48 h** | **72 h** |
|  |  | 4.31x10^5^ | 5.78x10^5^ | 2.23x10^6^ | 3.83x10^5^ | 4.65x10^5^ | 1.81x10^6^ | 11.1 | 19.6 | 18.8 |
