# Supplementary figures and images for: Effects of pressure on the survival and viability of cancer cells in vitro: An analytical study
Source: PLoS One. 2025 Feb 25;20(2):e0311685. doi: 10.1371/journal.pone.0311685 (PMC11856587; doi:10.1371/journal.pone.0311685)

**
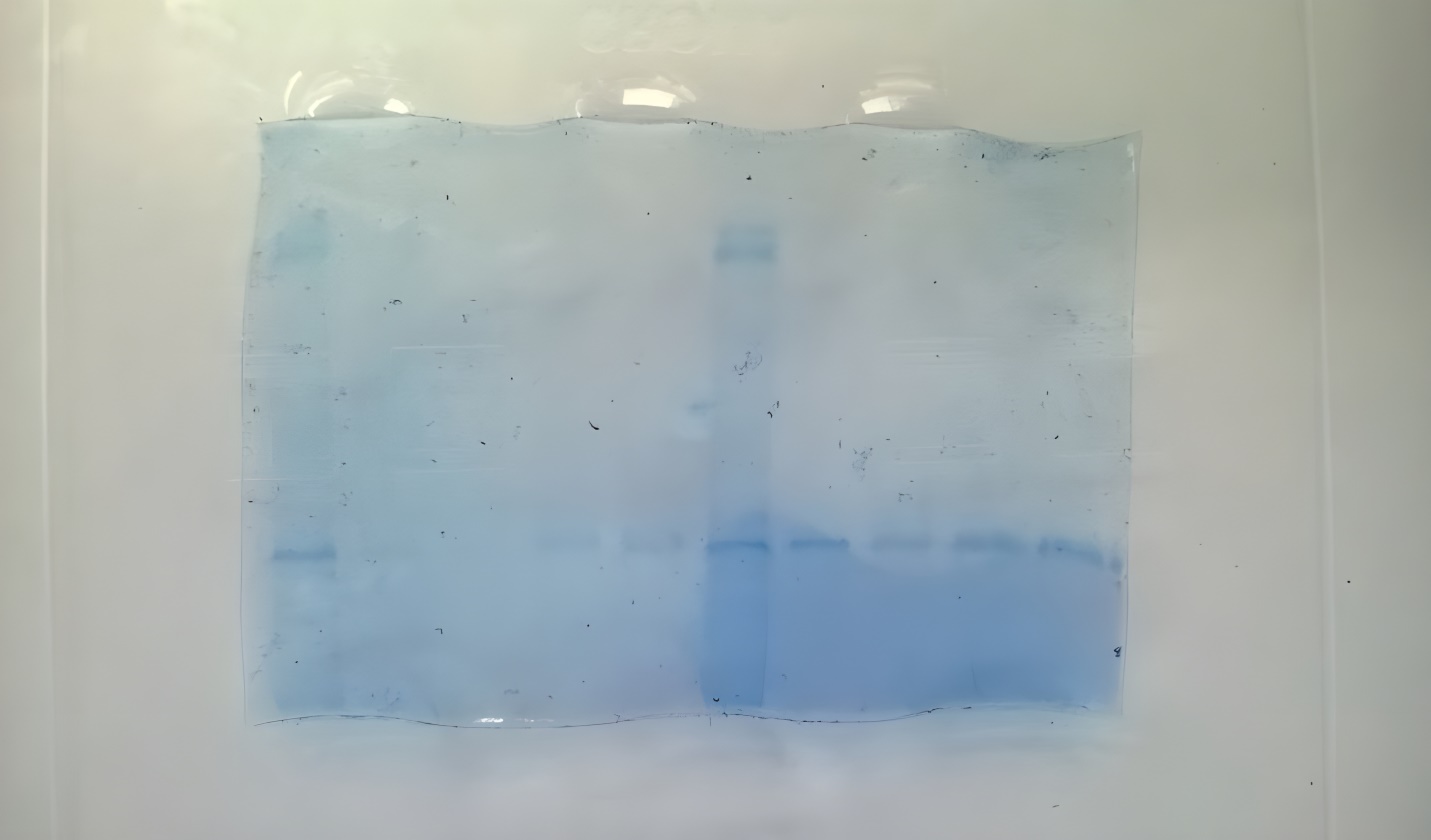
**

**Supplementary Figure 1.**

Supplement: S1 Fig — Lane 1: nuclear (0.65μg) 2: mitochondrial (0.08 μg) 3: microsomal (0.06 μg), 4,5: cytosolic (0.15 μg) fractions from MDA-MB-231 cells maintained at 14.7 psi; Lane 6: nuclear (1.5 μg) 7: mitochondrial (0.62 μg) 8: microsomal (0.45 μg), 9,10: cytosolic (0.50 μg) fractions from MDA-MB-231 cells maintained at 18 psi. (DOCX) [file pone.0311685.s003.docx]

**
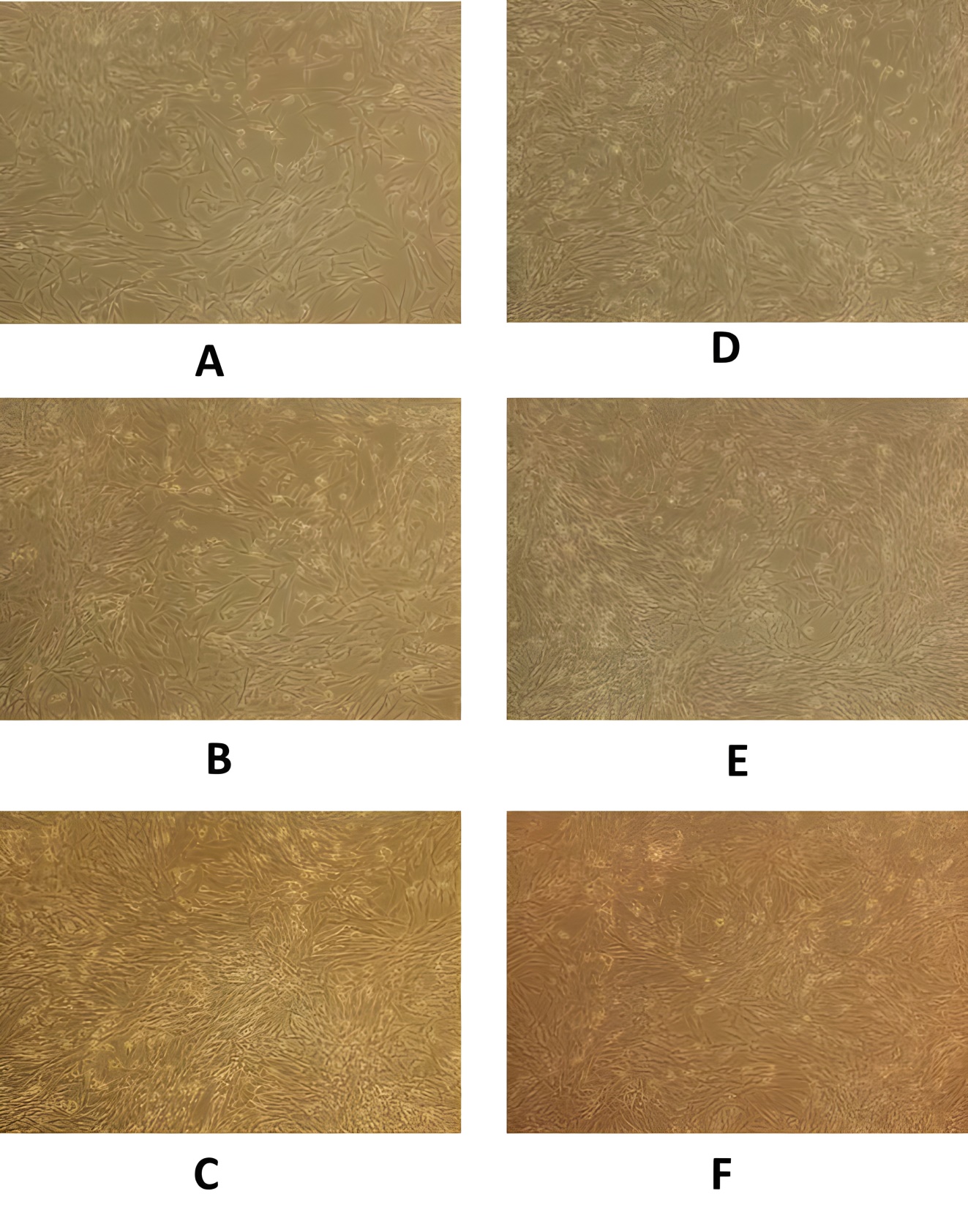
**

**Supplementary Figure 2. MDA-MB-231**

Supplement: S2 Fig — (DOCX) [file pone.0311685.s004.docx]

**
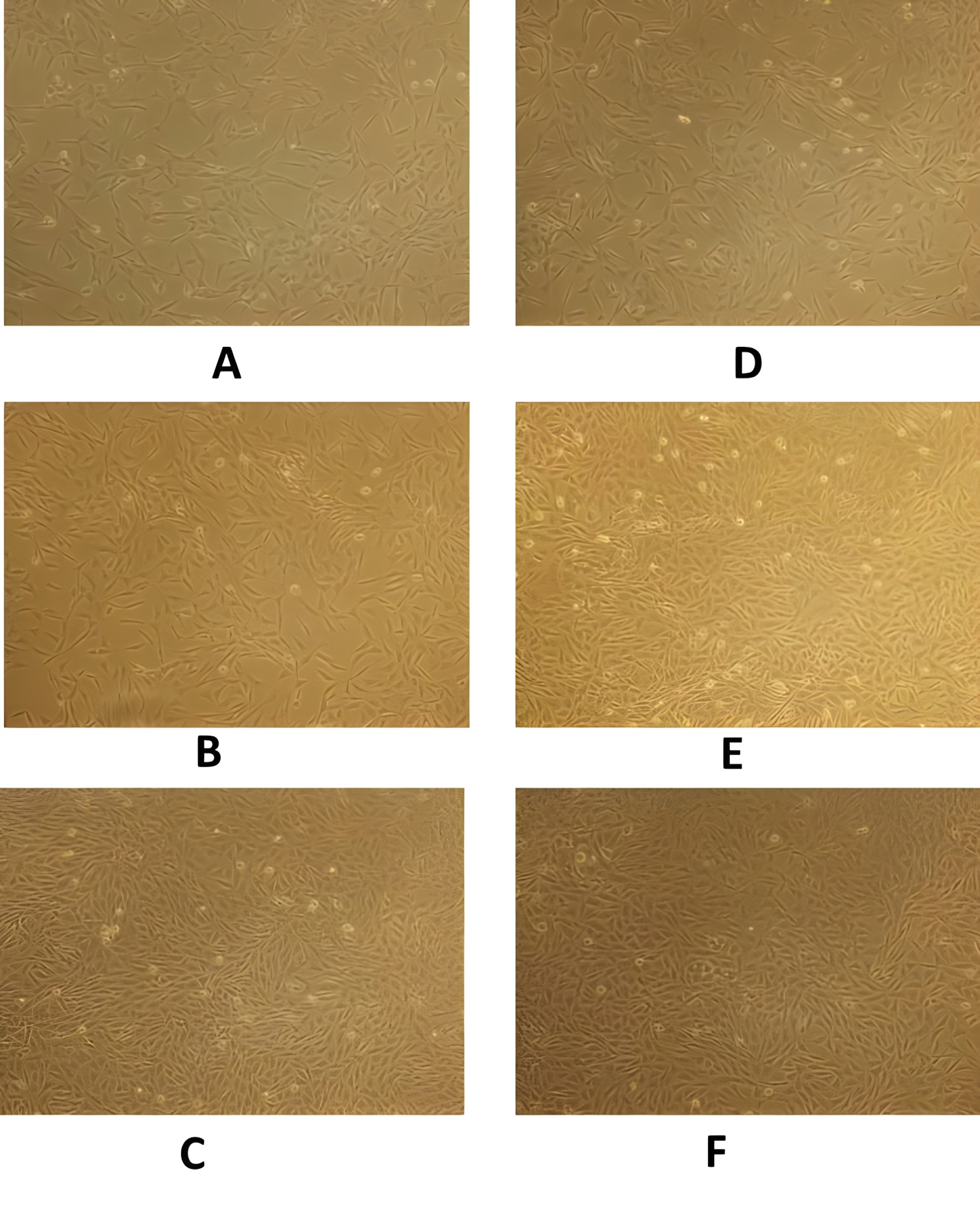
**

**Supplementary Figure 3. A549**

Supplement: S3 Fig — (DOCX) [file pone.0311685.s005.docx]
